# Changing trends in the prevalence of anencephaly in Liaoning province of Northeast China from 2006–2015: data from a population-based birth defects registry

**DOI:** 10.18632/oncotarget.17423

**Published:** 2017-04-26

**Authors:** Ting-Ting Gong, Qi-Jun Wu, Yan-Ling Chen, Cheng-Zhi Jiang, Da Li, Jing Li, Li-Li Li, Chen Zhou, Yan-Hong Huang

**Affiliations:** ^1^ Department of Obstetrics and Gynecology, Shengjing Hospital of China Medical University, Shenyang, China; ^2^ Department of Clinical Epidemiology, Shengjing Hospital of China Medical University, Shenyang, China; ^3^ Liaoning Women and Children’s Health Hospital, Shenyang, China; ^4^ School of Environmental and Chemical Engineering, Shenyang Ligong University, Shenyang, China; ^5^ Department of Science and Education, Shenyang Women and Children Health Care Centre, Shenyang, China; ^6^ Department of Children’s Health Prevention, Shenyang Women and Children Health Care Centre, Shenyang, China; ^7^ Department of Information Statistics, Shenyang Women and Children Health Care Centre, Shenyang, China

**Keywords:** anencephaly, Liaoning province, prevalence, time trend

## Abstract

The goal of this study was to assess the prevalence and trends of anencephaly on the basis of a large population-based cases identified by the Liaoning Birth Defects Registry, which included 14 cities over a 10-year period. Anencephaly prevalence, percent change, average changeand the contribution rates of each city were calculated. Statistical analysis was undertaken on the basis of a Poisson regression model. A total of 1600 anencephaly cases were collected during the observational period (4.92/10,000 live births). On average, the prevalence decreased 10.15% each year; this overall time trend was statistically significant (P<0.01). The top three leading cities were Huludao (10.33 per 10,000 live births), Chaoyang (8.56 per 10,000 live births) and Fuxin (6.36 per 10,000 live births). In contrast, Anshan (2.64 per 10,000 live births), Dalian (2.79 per 10,000 live births) and Yingkou (3.46 per 10,000 live births) were the cities with the lowest prevalence. Of note, significantly decreasing trends were observed in half of these cities (n=7). Additionally, Benxi, Yingkou and Dalian were the major cities contributing to over one third of the decreasing trend in Liaoning province. In conclusion, this study provided evidence of the decreasing prevalence of anencephaly from 2006 to 2015 in Liaoning province. In the future, prevention efforts should be strengthened to further reduce the risk of anencephaly in areas with high rates.

## INTRODUCTION

Anencephaly is defined as an absent calvarium with total or partial absence of the brain and includes cases of craniorachischisis. These result from neural tube closure defects by the 4^th^ week of pregnancy, or the 28^th^ day after conception [1]. Compared with spina bifida (7%) and encephalocele (46%), anencephaly has significantly higher mortality rate (100%) [2], and therefore, infants with this disease generally die during or shortly after birth. For this reason, anencephaly is a worldwide public health burden [3, 4]. Although previous studies have suggested that anencephaly is a multistep process strictly controlled by genes and a host of environmental factors, recently, there has been unequivocal evidence that a large proportion of anencephaly can be prevented by providing folic acid before pregnancy [5].

The prevalence of anencephaly varies over time and by region [6]. For instance, the prevalence in Northern Iran from 1998 to 2005 was 12 per 10,000 live births based on a cross-sectional hospital-based study published in 2010 [7]. In contrast, on the basis of the surveillance data from all births covered by the full member countries of the European Surveillance of Congenital Anomalies (EUROCAT), Obeid et al. [1] estimated the total prevalence of anencephaly between 2000 and 2010 was 3.52 per 10,000. A similar prevalence rate (2.81 per 10,000 births) was observed in Texas between 1999 and 2003 [8]. Compared with these countries, studies describing the time trend and prevalence of anencephaly in China on the basis of decade-old data demonstrated great variability in the reported prevalence rates. Between 2004 and 2005, the prevalence of anencephaly was 82.6 per 10,000 live births in Luliang Prefecture, one city in Shanxi province of China [9]. However, the prevalence was 4.2 per 10,000 live births in Guizhou province between 1996 and 2004 [10]. Although a recent report updated the data from Shanxi province, presenting a continuously decreasing trend that peaked at 40.0 per 10,000 live births in 2004 to a lower rate of 15.0 per 10,000 live births in 2014 [4]; similar reports investigating the situation in China on the basis of data from the recent decade have been limited. The prevalence of anencephaly over the recent decade as well as whether similar decreasing trends could still be observed in other cities is still unknown. Herein, we sought to address these aforementioned questions by evaluating anencephaly prevalence among infants in Liaoning province over 10-year period from 2006 to 2015.

## RESULTS

Table 1 shows the number of live births in 14 cities in Liaoning province during the 10-year observational period. Additionally, when comparing cities, Shenyang (the capital city of the province) had the largest number of live births in every year; in contrast, Benxi had the lowest numbers.

**Table 1:** The number of live births in each city in Liaoning province from 2006 to 2015

City	Year										Overall
	2006	2007	2008	2009	2010	2011	2012	2013	2014	2015	
Liaoning Province	306,734	341,432	330,414	321,353	307,826	304,079	353,108	321,171	364,400	298,437	3,248,954
Shenyang	52,256	61,108	59,196	59,200	57,521	58,335	69,721	67,854	80,997	65,118	631,306
Dalian	38,744	46,652	48,309	47,900	48,774	50,490	62,324	58,722	71,178	57,641	530,734
Anshan	29,270	31,305	29,647	27,721	25,184	25,603	28,790	25,855	36,171	20,798	280,344
Fushun	11,661	12,997	12,314	12,337	11,638	11,556	12,942	12,016	12,845	10,138	120,444
Benxi	8620	9435	8759	8842	8696	8261	9440	8700	9857	7627	88,237
Dandong	15,710	15,725	14,836	14,274	13,894	14,038	15,895	15,111	17,718	14,278	151,479
Jinzhou	24,293	24,261	23,149	22,342	21,255	20,098	22,559	20,860	16,137	16,985	211,939
Yingkou	16,987	18,924	19,667	19,070	17,947	18,484	21,309	14,224	21,684	16,515	184,811
Fuxin	14,158	14,142	13,353	13,322	12,370	11,800	13,050	9662	9121	11,752	122,730
Liaoyang	12,888	15,039	13,754	13,200	12,331	11,386	13,296	11,702	12,747	9251	125,594
Panjing	9887	9669	10,134	9009	8800	8867	10,362	9644	8276	9197	93,845
Tieling	21,263	20,298	21,456	19,854	18,421	16,945	18,938	14,960	17,389	15,269	184,793
Chaoyang	28,669	30,980	31,168	30,574	27,837	27,207	31,236	29,919	30,646	26,083	294,319
Huludao	22,328	30,897	24,672	23,708	23,158	21,009	23,246	21,942	19,634	17,785	228,379

**Figure 1:**
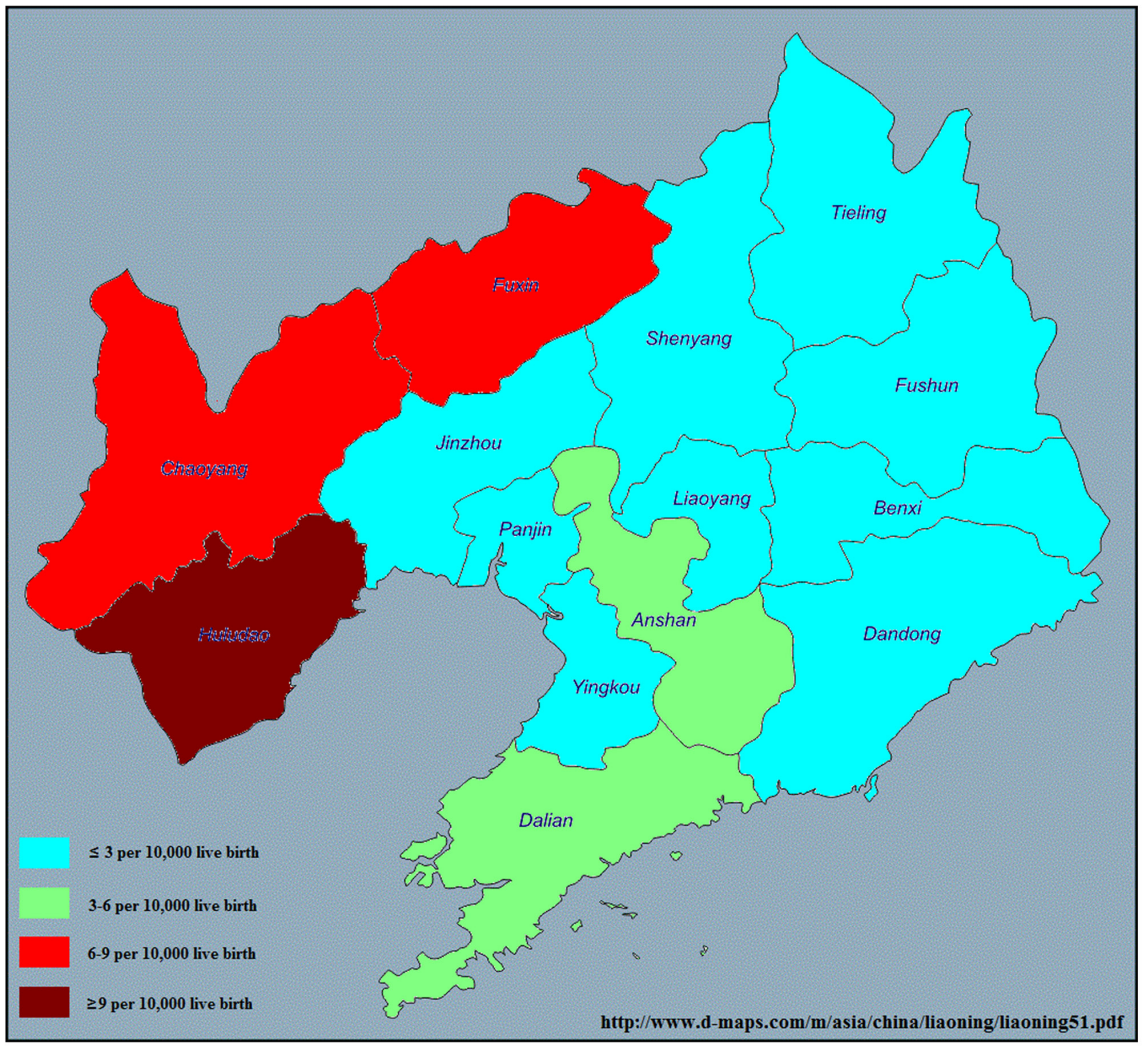
Prevalence of anencephaly of each city in Liaoning province from 2006–2015 www.d-maps.com/m/asia/china/liaoning/liaoning51.pdf

The prevalence of anencephaly in each city in Liaoning province is presented in Figure 1 and Table 2. Between 2006 and 2015, 1600 anencephaly cases were detected among 3,248,954 live births (4.92 per 10,000 live births). Huludao (10.33 per 10,000 live births), Chaoyang (8.56 per 10,000 live births) and Fuxin (6.36 per 10,000 live births) were the cities in Liaoning province with the highest rates, while, Anshan (2.64 per 10,000 live births), Dalian (2.79 per 10,000 live births) and Yingkou (3.46 per 10,000 live births) had the lowest anencephaly rates.

**Table 2:** The prevalence of anencephaly in each city in Liaoning province from 2006 to 2015 (per 10,000 live births)

City	Year										Overall
	2006	2007	2008	2009	2010	2011	2012	2013	2014	2015	
Liaoning Province	7.40	7.15	5.63	5.17	5.46	5.20	4.11	4.42	2.99	1.84	4.92
Shenyang	7.85	3.36	5.12	6.00	10.44	6.37	6.59	0.00	5.65	8.54	5.04
Dalian	10.64	4.93	3.51	3.08	10.60	7.00	6.18	4.23	11.31	3.32	2.79
Anshan	5.07	5.18	1.35	4.06	3.43	4.72	2.16	7.12	5.99	3.64	2.64
Fushun	4.90	4.18	3.25	1.62	9.05	1.40	4.92	6.29	3.75	1.52	4.07
Benxi	4.52	1.23	2.38	5.16	3.45	5.76	6.59	3.90	8.08	5.68	5.10
Dandong	4.29	2.38	5.47	4.33	4.84	4.27	6.97	4.87	6.78	6.15	4.42
Jinzhou	3.87	2.25	1.39	7.73	2.12	4.40	4.88	1.88	7.66	5.26	4.72
Yingkou	4.57	2.72	3.48	3.33	3.45	5.96	3.36	2.11	4.14	1.71	3.46
Fuxin	3.21	1.97	0.28	3.89	2.03	2.82	2.48	1.84	6.58	2.35	6.36
Liaoyang	2.76	0.87	0.48	0.99	1.31	1.40	1.77	1.82	2.55	0.00	3.90
Panjing	7.85	3.36	5.12	6.00	10.44	6.37	6.59	0.00	5.65	8.54	3.52
Tieling	10.64	4.93	3.51	3.08	10.60	7.00	6.18	4.23	11.31	3.32	4.71
Chaoyang	5.07	5.18	1.35	4.06	3.43	4.72	2.16	7.12	5.99	3.64	8.56
Huludao	4.90	4.18	3.25	1.62	9.05	1.40	4.92	6.29	3.75	1.52	10.33

**Figure 2:**
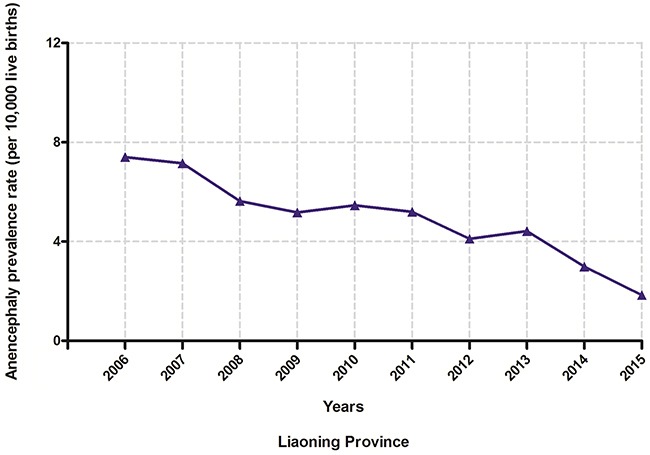
Trends in anencephaly prevalence rates (per 10,000 live births) in Liaoning province from 2006 to 2015

Figure 2 shows the rate and time trend of anencephaly prevalence in Liaoning province between 2006 and 2015. Overall, the prevalence decreased from 7.40 to 1.84 per 10,000 live births, or 10.15% per year (95% confidence interval (CI): −13.60% – −6.56%) (Table 3). When stratifying by cities, decreasing trends were observed in all cities, but these results were only statistically significant in seven cites (Table 3). Notably, Benxi, Yingkou and Dalian contributed over one third of the decreasing trend of anencephaly prevalence in Liaoning province (Table 4).

**Table 3:** Trends in anencephaly prevalence in each city of Liaoning from 2006 to 2015

Anencephaly	2006		2015		PC^†^ (%)	AC^†^ (%)	95% CI
	Case	Rate*	Case	Rate*			
Overall	227	7.40	55	1.84	−75.10	−10.15	−13.60, −6.56
Shenyang	41	7.85	18	2.76	−64.77	−12.01	−16.94, −6.79
Dalian	13	3.36	5	0.87	−74.15	−12.28	−19.82, −4.02
Anshan	15	5.12	1	0.48	−90.62	−7.60	−21.91, 9.35
Fushun	7	6.00	1	0.99	−83.57	−1.09	−13.08, 12.54
Benxi	9	10.44	1	1.31	−87.44	−18.62	−26.47, −9.93
Dandong	10	6.37	2	1.40	−77.99	−7.23	−15.79, 2.21
Jinzhou	16	6.59	3	1.77	−73.18	−7.04	−16.20, 3.12
Yingkou	0	0.00	3	1.82	N/A	−15.72	−24.37, −6.07
Fuxin	8	5.65	3	2.55	−54.82	−5.64	−14.74, 4.44
Liaoyang	11	8.54	0	0.00	−100.00	−7.78	−20.62, 7.13
Panjing	8	8.09	3	3.26	−59.69	−12.01	−18.27, −5.28
Tieling	12	5.64	3	1.96	−65.19	−4.02	−13.88, 6.97
Chaoyang	32	11.16	8	3.07	−72.52	−7.60	−12.77, −2.11
Huludao	45	20.15	4	2.25	−88.84	−11.57	−17.48, −5.24

AC, average change; CI, confidence interval; N/A, not available; PC, percent change.

* Anencephaly prevalence rates are expressed as the number per 10,000 live births.

† Percent change and average change between 2006 and 2015 was calculated based on the anencephaly prevalence rates.

**Table 4:** The relative contributions of each city in Liaoning province to the decreasing overall trend of anencephaly prevalence between 2006 and 2015

		Decreasing trend
City	β	Contribution rate (%)
Shenyang	−0.13	9.25
Dalian	−0.13	9.47
Anshan	−0.08	5.71
Fushun	−0.01	0.79
Benxi	−0.21	14.88
Dandong	−0.08	5.42
Jinzhou	−0.07	5.27
Yingkou	−0.17	12.36
Fuxin	−0.06	4.19
Liaoyang	−0.08	5.85
Panjing	−0.13	9.25
Tieling	−0.04	2.96
Chaoyang	−0.08	5.71
Huludao	−0.12	8.89

## DISCUSSION

To the best of our knowledge, this population-based report is one of the limited studies from China that describes the time trend of anencephaly prevalence on the basis of the data from most recent decade. Between 2006 and 2015, the prevalence of anencephaly in Liaoning province in Northeast China significantly decreased from 7.40 to 1.84 per 10,000 live births. Moreover, decreasing trends were observed in all 14 cities, but not all of them showed statistically significant changes. Since a high prevalence was still found in some cities, further prevention efforts are warranted to reduce the risk of anencephaly in these areas.

The present study found that the overall prevalence of anencephaly for Liaoning province from 2006 to 2015 was 4.92 per 10,000 live births. Geographic variation in the prevalence of this defect has been observed in different areas of different countries, which was proposed in a study describing the worldwide prevalence of neural tube defects including anencephaly [6]. The prevalence of anencephaly we found in this study was intermediate between the higher prevalence reported in Pakistan (113.3 per 10,000 live births) [11] in 2009 and the lower prevalence reported in Spain (0.3 per 10,000 live births) in 2012 [12]. Furthermore, a general decrease was observed in the median prevalence for anencephaly from the lower-middle to high income countries when stratified by country income [6]. Furthermore, compared to several reports in China, the prevalence was intermediate between the higher prevalence reported in Shanxi province (82.6 per 10,000 live births) [11] in 2009 and the lower prevalence reported in Guizhou Province (4.2 per 10,000 live births) [10] from 1996 to 2004.

Although termination of pregnancy for fetal anomaly has considerably reduced the prevalence of these anomalies in live births, it is certainly not an optimal solution for anencephaly, which is highly preventable with a readily available and low cost measure, namely, folic acid supplementation or food fortification [13]. Canada and the United States were the first countries to require mandatory fortification of enriched cereal grain products with 140 μg of folic acid per 100 g. Subsequently, many studies have observed a reduction in these anomalies after carrying out this policy [5]. As of the middle of 2012, 67 counties had fortified their wheat flour with folic acid (mandatory or voluntary programs) which affected over 2.2 billion people [5, 14]. Nevertheless, fortification has been uncommon in Asia and Europe, which might lead to the regional differences of prevalence rates [6]. In 2009, China initiated a nationwide folic acid supplementation program, and this program provides folic acid supplements free of charge to all women who have a rural registration as well as who plan to become pregnant [15]. In our study, we also noticed that the prevalence of anencephaly from 2012 to 2015 decreased dramatically from that of 2007 in the majority of these 14 cities, which may be partly attributable to the effects of this national policy. Nevertheless, not all of the 14 cities showed a statistically significant decreasing trend, suggesting that this phenomenon might be attributed to differences in the development of these cities and that fewer women take folic acid supplements before pregnancy in rural areas compared with urban areas in Northern China [16]. Therefore, policymakers are warranted to pay more attention to this issue in these cities and environments.

The present study had several strengths. The data used in this study were collected from a population-based birth defects registry with good quality control [17]. Of note, it included all 14 cities of Liaoning province over a relatively large time period (10 years), additionally, the most recent decade data up to 2015 comprehensively described the time trend and prevalence of anencephaly in Liaoning province. Despite these strengths, several limitations merit discussion. First, we had no access to the information regarding demographic factors of all live births in Liaoning province, which hindered our ability to evaluate the potential causes for the trends. Second, we could not get the prevalence of Liaoning province for any year prior to 2006. Hence, we failed to evaluate whether mandatory premarital physical check-ups, which became voluntary throughout the country on October 1, 2003, may have affected the time trend of anencephaly [4]. Additionally, under reporting could occur in the registry system, especially in developing countries. Third, the denominator of the prevalence of anencephaly was the total number of births (live births and still births at greater than or equal to 28 weeks of gestational age). Although inclusion of induced and spontaneous fetal deaths at less than 28 weeks of gestational age would more closely approximate the incidence of anencephaly, it is very impractical, as these pregnancy outcomes are often inaccurately counted compared to live and stillbirths [18]. Furthermore, compared with the number of live and stillbirths, the number of induced and spontaneous fetal deaths are small and unlikely to greatly affect these results [18]. Finally, the maximal diagnosis time for anencephaly cases was the seventh day after birth [17]. We did not include anencephaly cases confirmed after the seventh day in this study (n=4). Therefore, the calculated prevalence of anencephaly may be slightly lower than that which includes longer periods.

In summary, this population-based study provides recent and detailed evidence of the time trend and prevalence of anencephaly in Liaoning province between 2006 and 2015. The decreasing trend supports the notion that nationwide folic acid supplement programs may have been effective as a public health strategy to prevent anencephaly. However, it has been far effective when compared to mandatory folic acid fortification programs that have been carried out in developed countries. Notably, the prevalence of anencephaly remains high in some cities, which draws attention to the need to improve the efficiency of periconceptional folic acid supplementation. In the future, prevention efforts should be strengthened to reduce the risk of anencephaly in areas with a high prevalence rate.

## MATERIALS AND METHODS

### Study population and data source

Liaoning Women and Children’s Health Hospital is one of the sole obstetrical and gynecological hospitals for Liaoning province. It has also been a comprehensive care institution that has been in charge of women’s and children’s healthcare guidance. Data from 2006 to 2015 were retrieved from the maternal and child health certificate registry of Liaoning province, which is maintained by this hospital. Hospital-delivered live and stillbirth infants were included in this registry as the monitored subjects. This registry covers all 14 cities of the province (Shenyang, Dalian, Anshan, Fushun, Benxi, Dandong, Jinzhou, Yingkou, Fuxin, Liaoyang, Panjing, Tieling, Chaoyang and Huludao), with approximately 42 million inhabitants. The maximal diagnosis time for a congenital malformation case was the seventh day after birth [17].

The detailed procedures of data collection were described in previous report [17]. In brief, a “Birth Defects Register Form” was used to collect the related information on anencephalic infants. Once an anencephaly case was identified and confirmed at the monitored hospital, a trained obstetric or pediatric specialist would interview the mother of the infant to complete the aforementioned form. Subsequently, the “Birth Defects Register Form” was first submitted to the local maternal and child health facility and then to the provincial maternal and child health hospital, which is Liaoning Women and Children’s Health Hospital. For suspected anencephaly cases that were diagnosed through prenatal ultrasound scans, case ascertainment after termination or examination after the birth were requested. The data for these cases were reviewed and confirmed by a group of state-level experts in medical genetics and pediatrics [17].

According to the World Health Organization’s International Classification of Diseases, 10^th^ Revision, anencephaly is defined as an absent calvarium with total or partial absence of the brain and includes cases of craniorachischisis [13]. All cases of anencephaly were included in our analysis. The birth prevalence of anencephaly was expressed per 10,000 live births. The denominator was based exclusively on the total number of live births which were obtained primarily from the Liaoning Women and Children’s Health Hospital. Through this process we identified 1600 cases; the total number of live births in the study window was 3,248,954.

Data quality control was described in detail in a previous report [17]. Briefly, to ensure high quality data, the program manual dictates that diagnosis, data collection, data checking and medical records were verified by the expert group at each level. In addition, an independent retrospective survey was organized by the experts to find deficiencies and inaccuracies in the data [17]. This study was conducted in compliance with local and national regulations and was approved by the Institutional Review Board of Liaoning Women and Children’s Health Hospital.

### Statistical analysis

Anencephaly prevalence rates were calculated for nine 1-year time intervals from 2006 to 2015. To specifically look at time trends, a Poisson regression model was used to find the line of best fit for anencephaly prevalence by year, with year entered into the model as a continuous independent variable [19–22]. We used the method recommended by the National Cancer Institute to calculate the 95%CI of the average change [23]. The relative contributions for rate changes, which are used to determine the contributions individual cities made to the overall trend were calculated [24]. All analyses were conducted using SPSS for Windows (version 23; SPSS Inc., Chicago, IL, USA). *P*-values less than 0.05 were considered statistically significant.

## References

[1] Obeid R, Pietrzik K, Oakley GJ, Kancherla V, Holzgreve W, Wieser S (2015). Preventable spina bifida and anencephaly in Europe. Birth Defects Res A Clin Mol Teratol.

[2] Forrester MB, Merz RD (2003). First-year mortality rates for selected birth defects, Hawaii, 1986-1999. Am J Med Genet A.

[3] Yi Y, Lindemann M, Colligs A, Snowball C (2011). Economic burden of neural tube defects and impact of prevention with folic acid: a literature review. Eur J Pediatr.

[4] Liu J, Zhang L, Li Z, Jin L, Zhang Y, Ye R, Liu J, Ren A (2016). Prevalence and trend of neural tube defects in five counties in Shanxi province of Northern China, 2000 to 2014. Birth Defects Res A Clin Mol Teratol.

[5] Atta CA, Fiest KM, Frolkis AD, Jette N, Pringsheim T, St GC, Rajapakse T, Kaplan GG, Metcalfe A (2016). Global Birth Prevalence of Spina Bifida by Folic Acid Fortification Status: A Systematic Review and Meta-Analysis. Am J Public Health.

[6] Zaganjor I, Sekkarie A, Tsang BL, Williams J, Razzaghi H, Mulinare J, Sniezek JE, Cannon MJ, Rosenthal J (2016). Describing the Prevalence of Neural Tube Defects Worldwide: A Systematic Literature Review. PLoS One.

[7] Golalipour MJ, Najafi L, Keshtkar AA (2010). Prevalence of anencephaly in Gorgan, northern Iran. Arch Iran Med.

[8] Canfield MA, Marengo L, Ramadhani TA, Suarez L, Brender JD, Scheuerle A (2009). The prevalence and predictors of anencephaly and spina bifida in Texas. Paediatr Perinat Epidemiol.

[9] Chen G, Pei LJ, Huang J, Song XM, Lin LM, Gu X, Wu JX, Wang F, Wu JL, Chen JP, Liu JF, Xin RL, Zhang T (2009). Unusual patterns of neural tube defects in a high risk region of northern China. Biomed Environ Sci.

[10] Liu J, Yang GZ, Zhou JL, Cao SP, Chau DH, Kung HF, Lin MC (2007). Prevalence of neural tube defects in economically and socially deprived area of China. Child’s Nervous System.

[11] Jentink J, Dolk H, Loane MA, Morris JK, Wellesley D, Garne E, de Jong-van DBL (2010). Intrauterine exposure to carbamazepine and specific congenital malformations: systematic review and case-control study. BMJ.

[12] European Surveillance of Congenital Anomalies (EUROCAT) (2012). Prevalence Tables. http://www.eurocat-network.eu/accessprevalencedata.

[13] Khoshnood B, Loane M, de Walle H, Arriola L, Addor MC, Barisic I, Beres J, Bianchi F, Dias C, Draper E, Garne E, Gatt M, Haeusler M (2015). Long term trends in prevalence of neural tube defects in Europe: population based study. BMJ.

[14] Food Fortification Initiative Enhancing Grains for Healthier Lives. Country profiles. http://www.ffinetwork.org/country_profiles/index.php.

[15] Ren AG (2015). Prevention of neural tube defects with folic acid: The Chinese experience. World J Clin Pediatr.

[16] Liu J, Jin L, Meng Q, Gao L, Zhang L, Li Z, Ren A (2015). Changes in folic acid supplementation behaviour among women of reproductive age after the implementation of a massive supplementation programme in China. Public Health Nutr.

[17] Xu L, Li X, Dai L, Yuan X, Liang J, Zhou G, Li Q, He C, Miao L, Wang Y, Zhu J (2011). Assessing the trend of gastroschisis prevalence in China from 1996 to 2007 using two analytical methods. Birth Defects Res A Clin Mol Teratol.

[18] Li X, Zhu J, Wang Y, Mu D, Dai L, Zhou G, Li Q, Wang H, Li M, Liang J (2013). Geographic and urban-rural disparities in the total prevalence of neural tube defects and their subtypes during 2006-2008 in China: a study using the hospital-based birth defects surveillance system. Bmc Public Health.

[19] Wu QJ, Li LL, Li J, Zhou C, Huang YH (2016). Time trends of neonatal mortality by causes of death in Shenyang, 1997-2014. Oncotarget.

[20] Huang YH, Wu QJ, Li LL, Li D, Li J, Zhou C, Wu L, Zhu J, Gong TT (2016). Different extent in decline of infant mortality by region and cause in Shenyang, China. Sci Rep.

[21] Gong TT, Wu QJ, Chen YL, Jiang CZ, Li J, Li LL, Liu CX, Li D, Zhou C, Huang YH (2016). Evaluating the time trends in prevalence of exomphalos in 14 cities of Liaoning province, 2006 to 2015. Sci Rep.

[22] Li N, Chen YL, Li J, Li LL, Jiang CZ, Zhou C, Liu CX, Li D, Gong TT, Wu QJ, Huang YH (2016). Decreasing prevalence and time trend of gastroschisis in 14 cities of Liaoning Province: 2006-2015. Sci Rep.

[23] Hankey BF, Ries LA, Kosary CL, Feuer EJ, Merrill RM, Clegg LX, Edwards BK (2000). Partitioning linear trends in age-adjusted rates. Cancer Causes Control.

[24] Wu QJ, Vogtmann E, Zhang W, Xie L, Yang WS, Tan YT, Gao J, Xiang YB (2012). Cancer incidence among adolescents and young adults in urban Shanghai, 1973-2005. PloS One.

